# Diagnostic accuracy of Magnetic Resonance Imaging in assessment of Meniscal and ACL tear: Correlation with arthroscopy

**DOI:** 10.12669/pjms.312.6499

**Published:** 2015

**Authors:** Jamal Yaqoob, Muhammad Shahbaz Alam, Nadeem Khalid

**Affiliations:** 1 Dr. Jamal Yaqoob, MBBS, MCPS, FCPS, FRCR. Consultant Radiologist, Department of Radiology, Dallah Hospital, Riyadh, Kingdom of Saudi Arabia; 2 Dr. Muhammad Shahbaz Alam, MBBS, FCPS. Specialist Radiologist, Department of Radiology, Dallah Hospital, Riyadh, Kingdom of Saudi Arabia; 3 Dr. Nadeem Khalid, MBBS, MSc, FRCS. Consultant Orthopedic Surgeon, Department of Orthopedics, Dallah Hospital, Riyadh, Kingdom of Saudi Arabia

**Keywords:** Magnetic Resonance Imaging (MRI), Medial Meniscus, Lateral Meniscus, Anterior Cruciate Ligament (ACL), Posterior Cruciate Ligament (PCL)

## Abstract

**Objective:**

To determine the diagnostic accuracy of magnetic resonance imaging (MRI) in injuries related to anterior cruciate ligament and menisci and compare its effectiveness with that of arthroscopy.

**Methods:**

This retrospective cross-sectional study was conducted in the department of Radiology & Medical Imaging of Dallah Hospital, Riyadh, Kingdom of Saudi Arabia from September 2012 to March 2014. Fifty four patients (including 30 men and 24 women) with internal derangement of knee referred from the orthopedic consulting clinics underwent MR imaging followed by arthroscopic evaluation. The presence of meniscal and ligamentous abnormality on the imaging was documented by two trained radiologist. Findings were later compared with arthroscopic findings.

**Results:**

The sensitivity, specificity and accuracy of MR imaging for menisci and ACL injury were calculated: 100% sensitivity, 88.4% specificity, 90% positive predictive value, 100% negative predictive value, and 94.4% accuracy were noted for medial meniscal injury. Similarly, MR had sensitivity of 85.7%, specificity of 95%, positive predictive value of 85.7%, negative predictive value of 95%, and accuracy of 92.5% for lateral meniscal injuries. Likewise, anterior cruciate ligament had 91.6% sensitivity, 95.2% specificity, 84.6% positive predictive value, 97.5% negative predictive value, and 94.4% accuracy.

**Conclusion:**

MRI is extremely helpful in identifying meniscal and anterior cruciate ligaments tears. MR imaging has high negative predictive value making it better choice as screening tool compared to diagnostic arthroscopic evaluation in most patients with soft tissue trauma to knee.

## INTRODUCTION

Magnetic Resonance Imaging (MRI) has now established itself as fast and non-invasive imaging alternative complementing physical examination in the evaluation of injuries of the knee. Although conventional radiography and computed tomography (CT) are frequently used for detection of osseous injuries of the knee, MRI with its much better soft tissue contrast remains the main imaging modality of excellence for accurately depicting abnormalities of articular cartilage and soft tissue injuries of tendons, ligaments, and the menisci.^1^

According to early reports, the prospect of MRI in evaluating the knee joint was first delineated by Kean, Moon and coworkers way back in 1983.^2^ Since then, because of its improved accuracy, MRI has markedly expanded its contribution in evaluation of various types of knee injuries. The knee has become the most frequently studied articulation with MRI considered an alternative to diagnostic arthroscopy in assessing internal structures of knee i.e. menisci and cruciate ligaments. Injuries to menisci and cruciate ligaments detected with MRI have higher sensitivity and specificity in comparison with arthroscopy, which is still viewed as the standard of reference. Needless diagnostic arthroscopies can be evaded by MRI and can also aid orthopedic surgeon in surgical planning leading to decreased procedural time. Imaging in multiple planes allows assessment of all relevant abnormalities of the knee joints in short span of time and incurring no radiation dose to the patient.^3^

Although there are number of studies done world-wide depicting accuracy of MRI in meniscal and ligamentous pathologies, however, data in our part is scarce and limited. So, the purpose of this study was to look for the diagnostic performance of the MRI in the evaluation of menisci and cruciate ligaments in local population and compare it with arthroscopy which is currently regarded as the reference point. Moreover, the validity of MRI in predicting difference in medial and lateral meniscal injuries was also studied.

## METHODS

This retrospective study from September 2012 to March 2014 included 54 adult patients with history of knee pain related to previous trauma to the knee referred from the orthopedic consulting clinic for MRI examination. All 54 patients also underwent arthroscopic evaluation for menisci and cruciate injuries based on clinical suspicion by single orthopedic surgeon. The total number of patients consisted of 30 men and 24 women with age ranging from 19-59 years. (Mean age: 30.4 years) The shortest interval between MRI and arthroscopy was 5 days and longest was 65 days. Ethics committee approval was obtained from the institutional review board of Dallah Hospital. Because our study was retrospective, no informed consent was obtained.

MRI of the affected knee was performed on either of the two 1.5 tesla scanners: Siemens, Magnetom Avanto (25 patients) and General Electric Medical Systems, Optima MR 450 w (29 patients). The imaging protocol included sagittal T1, T2, GRE (Gradient Echo); coronal T2, PD (Proton Density) and axial T2 * GRE sequences. Fat suppression (FS) was obtained in all cases with T2 and PD sequences as our new departmental imaging protocol. Dedicated extremity knee coil used in all cases. Imaging parameters were field of view of 14-16 cm; 320 ×240 matrix sizes; slice thickness of 3.0 mm; an intersection gap of 1 mm for both sagittal and coronal images. The total time taken to perform the MRI examination including in initial survey sequence was around 25 minutes. Exclusion criteria included postoperative patients, previously identified cases of ligamentous injuries and those patients with contraindications to MRI such as claustrophobia, pregnancy and patients having metallic implants.

Interpretations of the cases were performed 2 to 3 months after acquisition of images to prevent possible reviewer bias. The MR scans were reviewed by two trained and qualified radiologists, each having at least five years experience in musculoskeletal MR imaging. Both reviewers were unaware of the interpretations of each other and arthroscopic finding to maintain objectivity. Findings were compared with arthroscopic reports. The average interval between MRI and arthroscopy was approximately 27 days (median 21 days, range 1-120 days). Arthroscopic evaluation was considered as gold standard.

### Data analysis

Difference in performance between the two reviewers was tested for significance by using kappa statistics. Kappa value of greater than 0.75 indicated excellent agreement. Confidence interval measures also showed no significant difference (p >0.05) between the interpretation of two reviewers. (Table-I)

**Table-I:** Agreement between reviewer’s interpretation of MR images.

Reviewer	Lateral Meniscus	Medial Meniscus	ACL	Overall
1	0.923	0.954	0.961	0.938
2	0.948	0.954	0.956	0.953

*Note:* Kappa value of greater than 0.75 indicates excellent agreement.

Meniscal injuries on MRI were scored according to a grading system described by Lotysch et al.^4^ and Crues et al.^5^ Grade-3 signal intensity on MRI was defined as abnormal signals in meniscus extending to the articular surface. A single abnormal image was considered sufficient for diagnosing a meniscus as torn on MRI. Grade-1 and 2 signal changes in meniscus not reaching the articular surface were not considered tears. Normal ACL appearance was of a group of fibers of predominantly of hypo intense or intermediate signal intensity on both sagittal and coronal images. Partially torn ligament appeared as abnormal signal intensity with indistinctness or wavy appearance of fibers on sagittal and coronal images. Likewise, non-visualization or discontinuity of fibres was considered as full thickness or complete tear on MRI.^6^ Normal and partially torn ligaments were classified as one group and complete tear as another group for the sake of statistical analysis.

All arthroscopic procedures were done by an experienced orthopedic surgeon with more than 10 years experience in knee arthroscopy. The arthroscope, which had a 70 degree viewing angle, was introduced into the knee through standard anteromedial and anterolateral portals. After performing the diagnostic arthroscopy decision on continuing further with therapeutic intervention was taken by the orthopedic surgeon, if required. All the data was collected on a proforma and entered on SPSS computer program (Version 19). The presence of meniscal and ligamentous injuries were noted and further comparison were made with arthroscopic findings.

Sensitivity, specificity, positive predictive value (PPV), negative predictive value (NPV) and accuracy of MRI were calculated considering arthroscopy as gold standard.

## RESULTS

On arthroscopy, medial meniscus tears were found in 26 patients (48%), lateral meniscus tears were found in 9 patients (16.6%), both menisci were torn in 5 patients (9%), and no meniscal injury was found in 14 patients (26%). Out of the total 54 cases, 16 patients (30%) showed meniscal injury alone.

In 8 patients (15%), both ACL and meniscus tears were noted. Isolated ACL tears were seen in 3 patients (5%) Discontinuity of ACL was seen in 5 patients and non-visualization of ACL in 6 patients. Only 2 patients (3.7%) had PCL tears. Collateral ligament tears were not found in our study. In 39 patients (72%) there was right knee involvement, while in 15 patients (28%) left knee was the affected knee.

**Fig.1:**
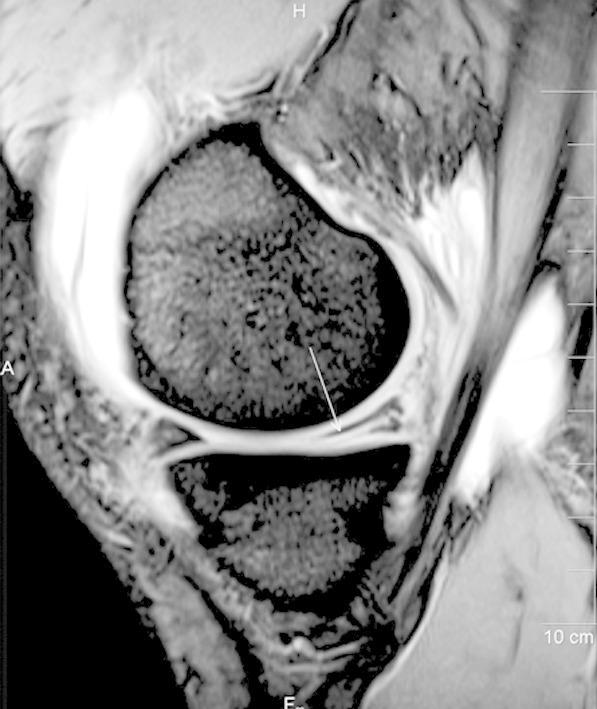
Sagittal T2-weighted MR image revealing grade-3 tear in the posterior horn of medial meniscus. Linear hyperintense signal (arrow) seen extending to the inferior articular surface. Joint effusion also seen in the image.

Out of the 31 patients of medial meniscal injury, grade-3 tear was detected on MRI in 18 patients (58%) while grade-2 and 1 signals were seen in 8 (26%) and 5 (16%) patients respectively. The posterior horn of the medial meniscus was most frequently injured in our study as involvement was seen in 22 patients (71%). (Fig.1)

Bucket handle tear of medial meniscus were confirmed on arthroscopy in 3 patients identified as double PCL sign on MRI. Lateral meniscal injuries were noted in 14 patients. Grade-3 tear was observed in 2 patients (14%), while grade-2 and 1 signals were seen in 9 (64%) and 3 (21%) patients respectively. The posterior horn involvement was seen in 6 (43%) of lateral menisci. Two lateral menisci were identified of discoid morphology. Results obtained after comparing arthroscopic and MRI findings are listed in Table-II.

**Table-II:** Accuracy of MRI in diagnosing Medial meniscus, Lateral meniscus, ACL injuries.

	Sensitivity	Specificity	Positive Predictive Value	Negative Predictive Value	Accuracy
Medial Meniscus	100%(28/28+0)	88.4%(23/23+3)	90%(28/31)	100(3/3+0)	94.4%(28+23/54)
Lateral Meniscus	85.7%(12/12+2)	95%%(38/38+2)	85.7%(12/14)	95%(38/38+2)	92.5%(12+38/54)
ACL	91.6%(11/11+1)	95.2%(40/40+2)	84.6%(11/13)	97.5%(40/40+1)	94.4%(11+40/54)

**Fig.2:**
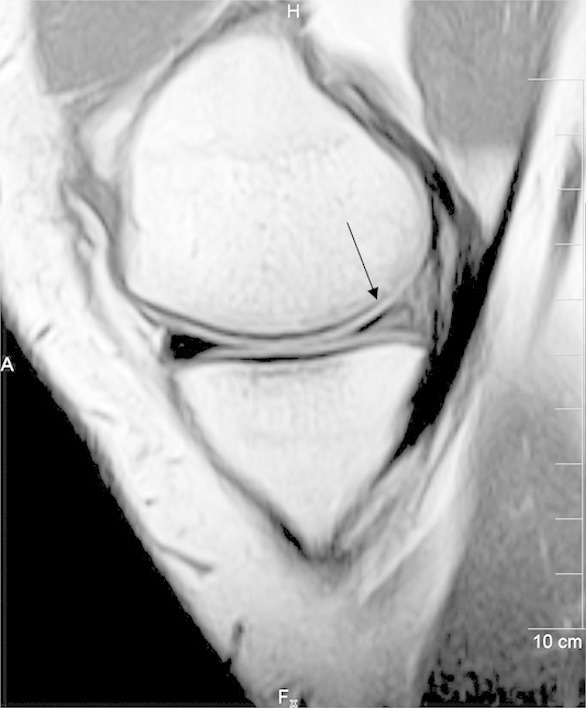
Sagittal T1-weighted MR image showing Grade-3a tear in the posterior horn of medial meniscus that extends to the inferior articular surface. (Black arrow) On arthroscopy the tear was not documented performed 14 days after MR imaging.

The results of our study match the sensitivity, specificity and accuracy of MRI for detection of meniscal and ACL injuries reported in earlier studies.^7^ Meniscal tears on MRI were equivocal in 2 of the 54 knees. The signal was horizontal in the middle portion of the meniscus and extended near the inferior meniscal surface in both cases. Location was posterior horn of the medial meniscus in these two cases. (Fig.2)

On arthroscopy no tear was identified in either patient. These equivocal tears (Grade-3a) as they are classified have a prevalence of about 14%.^8^ Therefore, diagnosis of a tear should be made only when definitive findings of high signal intensity are seen reaching the articular surface.

In 19 out of the 20 patients with confirmed grade-3 tears on arthroscopy, abnormal signals reaching the articular surface were seen on two consecutive MR sections. Only one patient with confirmed grade-3 tear on arthroscopy showed abnormal signals on single MR slice and not on two consecutive images. Since the number of patients with grade-3 tears was small statistical values were not calculated for tears diagnosed by two consecutive MR sections as variations were expected by random chance in the calculation of accuracy values.

## DISCUSSION

MRI is the non-invasive imaging modality of choice in assessing knee abnormalities. The higher negative predictive value and higher specificity endorse the use of MRI as a screening tool, hence facilitating in evading needless arthroscopies.^9^ MRI and clinical examination both provide accurate non-invasive information for diagnosing menisci and the ACL injuries.^10^ Diagnostic arthroscopy is used to clarify doubtful cases of meniscal tears. Unfortunately, it is an invasive procedure with possible problems and risk to the patient. Its overuse can result in unnecessary complications, such as sephenous and peroneal nerve injuries, superficial and deep infections, vascular injuries and pulmonary embolism.^11^

Recently there has been significant increase in the usage of MRI, subsequently diagnostic arthroscopies have reduced. In USA alone, there was a 144% rise in MR imaging of the knee between years 1993 and 1999. This resulted in considerable decline in diagnostic arthroscopy procedures by 54%, whereas therapeutic arthroscopies increased by 27%.^12^

The use of MRI to establish or confirm a diagnosis of meniscal tear has become a routine practice, and the accuracy of this imaging modality for diagnosing meniscal tears has been extensively reported in the literature.^13^ In our study medial meniscus tears were more common (36.7%) than lateral meniscus. (17.3%) Frequent involvement of posterior horn of medial meniscus and anterior horn of lateral meniscus observed favoured other earlier studies.^14^ Grade-3 tear was most common followed by Grade-2 and grade-1. High signal intensity seen in meniscal degeneration was due to absorbed synovial fluid. We in our study found that both sagittal and coronal planes helpful in meniscal evaluation. Moreover, T2* weighted GRE images better showed the meniscal tears than the FSE images, as reported earlier by Rubin et al.^15^

Mackenzie R et al.^16^ described the overall sensitivity of MRI for picking up menisci and cruciate ligament injuries to be 88% with overall specificity 94% when correlated with arthroscopic evaluation. Excellent correlation was found between MRI and arthroscopy in our study as results were comparable to previous studies in literature.

Similarly, a systematic meta-analysis of 29 studies done by Oei et al.^17^ described meniscal and cruciate ligament injuries in 3683 knees in the years 1991-2000. Using very strict inclusion and exclusion criteria, Oei et al.^17^ found pooled sensitivity and specificities for medial meniscus and lateral meniscus of 93%, 88% and 79%, 95% respectively. For ACL and PCL tears, collective sensitivities and specificities were 94%, 91% and 94%, 99% respectively.

**Fig.3:**
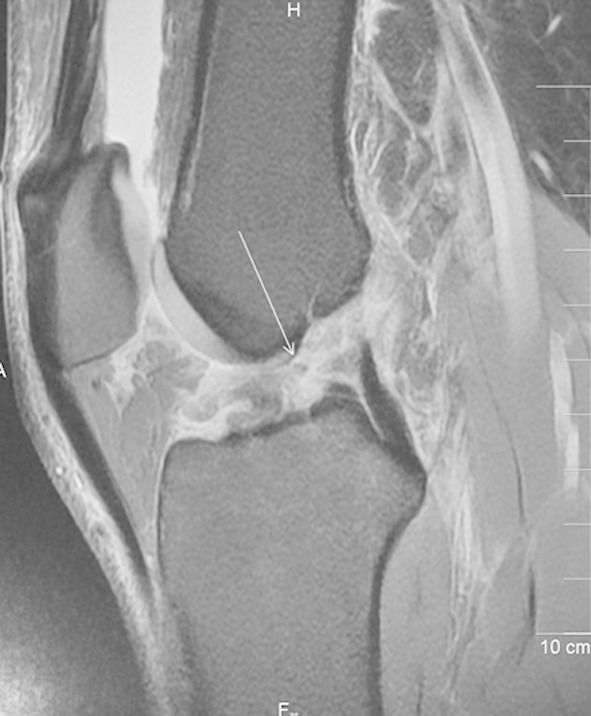
Sagittal TE weighted MR image reveal what was thought to be complete ACL rupture (arrow) was not appreciated as a complete rupture at arthroscopy. According to the arthroscopist it was a partial tear that involved approximately 75% of the ligamentous body.

Partial tears of the ACL are considered challenging for the radiologist and the orthopedic surgeon to explain in common terms. (Fig.3)

The arthroscopic characterization of partial tear is variable and ranges from the presence of some interrupted fibers to a sub totally ruptured ACL. In our study we adopted the approach used by Rubin et al.^18^ that is to distinguish complete ACL tear from the rest. Therefore a complete ACL tear on MRI was seen in 11 patients (20%) Discontinuity and non-visualization of ACL fibres were considered predictors of a complete ACL tear. Only 5% of ACL tears were identified in isolation, while 15% were associated with meniscal tears. ACL tear with mid-substance hyperintense signal was seen in 5 patients (9.2%) while non-visualization of the ACL was identified in 6 patients (11%). We found sagittal T2-weighted images evaluating ACL abnormality with great degree of accuracy, while coronal T2-weighted and PD sequences were helpful in the evaluation of the proximal and distal ACL attachment sites.

**Fig.4:**
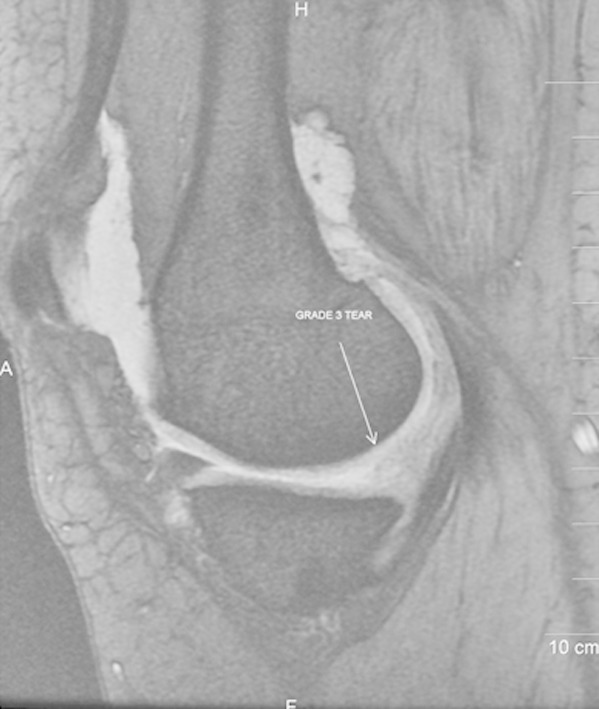
Sagittal T2-weighted MR image revealing complex meniscal tear in the posterior horn of medial meniscus seen extending to the articular surfaces. The tear was not recognized at arthroscopy performed 16 days after MR imaging and therefore constituting a false-positive diagnosis at MR.

In patients with ACL tear subtle peripheral tears may be present in both lateral and medial menisci which should be carefully looked for on imaging, especially posterior horn of medial meniscus. Similarly the inferior surface of menisci is problematic area to examine arthroscopically, and requires expertise of the arthroscopist in the accuracy of the procedure. The specificity decreases if a tear is diagnosed when there are only equivocal or probable findings on MRI because these findings usually do not represent a tear at arthroscopic examination. (Fig.4)

**Fig.5:**
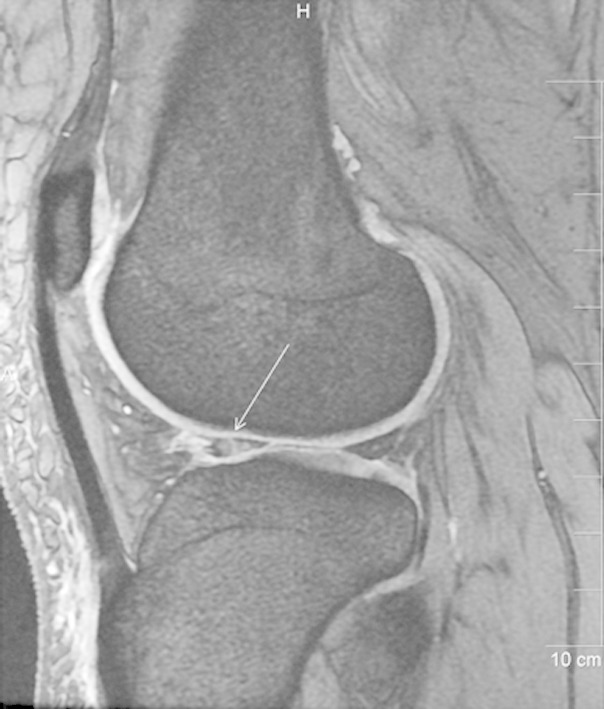
Sagittal T2-weighted MR image reveals tear in the anterior horn of lateral meniscus that appears globular signal of increased intensity extending to superior and inferior articular surfaces. (arrow) The tear was not recognized at arthroscopy performed 7 days after MR imaging and therefore constituting a false-positive diagnosis at MR.

In particular, abnormalities involving the free edge of the body of the lateral meniscus should not be overlooked as tears to prevent unnecessary surgical intervention.^19^,^20^ Another potential explanation for false-positive result in evaluation of meniscal tears is globular or amorphous area of abnormal signal intensity that contacts an articular surface but is less distinct without definite linear component. Such meniscal lesions are usually seen in the setting of acute trauma and are considered transient, referred to as meniscal contusion. The pathophysiology of this injury is not known. However, the non existence of a meniscal tear on arthroscopy and lack of progression on follow-up imaging suggests that the prognosis for meniscal contusion is much favourable than it is for complete meniscal tear.^21^ The meniscal contusional injury was the likely explanation for the two lateral meniscal false positive cases (Fig.5).

Our study did not specifically compare various types of meniscal tears, as arthroscopic details did not identify the precise type of meniscal lesion. PCL tear was identified in only 2 patients (3.7%) appearing as hyperintense signal in one patient and ligament discontinuity in the other patient. PCL is difficult to visualize during arthroscopy with intact ACL, and in such cases physical examination is often performed under cover of anesthesia to determine rupture of PCL. MRI findings preceding arthroscopic evaluation are often helpful in PCL evaluation.^22^ In our study PCL rupture was identified in both patients by arthroscopy.

MRI systems used in this study had 1.5 tesla field strength considered appropriate for producing diagnostic images of high quality. Magee et al.^23^ established that MRI of the knee performed at 3.0 tesla compares favorably in sensitivity and specificity with studies performed at 1.5 tesla or lower field strength scanners. However their study did not compare directly between different field strengths in one study population. Therefore, further studies may be needed to determine the true diagnostic performance of different field strength scanners. There is scope for further research using different MRI sequences so as to find out the best technique which can be used as a standard protocol for imaging various structures of the knee while keeping the examination within reasonable time and cost.

The design of our study had several limitations. Firstly it was retrospective study on small number of patients limiting our ability to statistically correlate the specific MRI findings to the presence or absence of a tear. Secondly the study did not define precisely which diagnosis at MRI indicated a need for arthroscopy. Thirdly MRI imaging was done in selected patients undergoing arthroscopic evaluation thus overrating the sensitivity and underrating the specificity, because not all patients with negative MR imaging underwent arthroscopy as well. There was inherent referral bias because only patients referred for MRI were included in the study. Inherent verification bias affected all patients as they all had undergone MRI before arthroscopy likely influencing the decision to perform arthroscopy. Context bias was also possible because the MRI readers were aware that all patients had undergone arthroscopy, which may have increased the likelihood of the readers interpreting an abnormality as a tear.

## CONCLUSION

Our study revealed MRI having high sensitivity, specificity and accuracy for meniscal and ligament injuries of the knee joint. Results of the present study are consistent with earlier larger studies, therefore there is substantial evidence to conclude that MRI is highly accurate in diagnosing meniscal and ACL tears. MRI is now commonly used before diagnostic arthroscopy in most settings, and is considered an effective screening tool in most patients because it is faster, non-invasive and does not involve morbidity associated with arthroscopy. MRI findings before arthroscopy help in the management of meniscal and ligament injuries, ultimately improving patient outcome.
